# First report of tethered cord syndrome in a patient with Sotos syndrome

**DOI:** 10.1186/s12887-020-02068-y

**Published:** 2020-04-24

**Authors:** Pelin Kuzucu, Tolga Türkmen, Alp Özgün Börcek

**Affiliations:** Division of Pediatric Neurosurgery, Gazi University Faculty of Medicine, Gazi Üniversitesi. Beyin ve Sinir Cerrahisi. C blok. 1 Kat., Yenimahalle, 06760 Ankara, Turkey

**Keywords:** Sacral lipoma, Filum terminale, Sotos syndrome, Spina bifida, Tethered cord syndrome

## Abstract

**Background:**

Sotos syndrome is caused by a gene deletion with an autosomal dominant pattern of inheritance. The Sotos syndrome was first described by Juan Sotos. Cole and Hughes identified the clinical characteristics of this syndrome. This syndrome is characterized by macrocephaly, frontal bossing, ocular hypertelorism, overgrowth, subdural hygroma, ventricular dilatation, agenesis of the corpus callosum. This syndrome is associated with mutations in NSD 1 (nuclear receptor SET domain-containing protein 1) gene, protein insufficiency, and a 5q35 microdeletion. Sotos syndrome is reported to occur in approximately 1/10,000–15,000 births.

**Case presentation:**

We present a patient with Sotos syndrome who is harboring a sacral lipoma and tethered cord syndrome and she had growth retardation, frontal bossing and hypertelorism. After a standard approach for tethered cord syndrome, the patient was discharged 3 days after without any additional neurodeficits.

**Conclusion:**

In the literature, sacral lipoma and tethered cord syndrome with Sotos syndrome have not been published yet.

## Background

The Sotos syndrome was first described by Juan Sotos in 1964 [1]. Cole and Hughes identified the clinical characteristics of this syndrome, in 1994. This syndrome is characterized by general overgrowth, coarse face, superciliary arches, large and long head with the high bossed forehead, hypertelorism, a square and pointed chin and macrocephaly [2] kyphoscoliosis, and cardiac defects [3]. Neuroradiologic manifestations are hydrocephalus, subdural hygroma, ventricular dilatation, agenesis of the corpus callosum, mega cisterna magna, underdevelopment of the cerebellar vermis [4], mental retardation, kyphoscoliosis, and cardiac defects [3].

Sotos syndrome occurs in approximately 1/10,000–1/15,000 live births with an autosomal dominant pattern of inheritance [5]. In approximately 90% of the patients, Sotos syndrome is associated with mutations in NSD 1 (nuclear receptor SET domain-containing protein 1) gene, protein insufficiency and a 5q35 microdeletion [5].

Although the syndrome is associated with various neural developmental anomalies, to date there is no report in the literature relating to Sotos syndrome with tethered cord syndrome.

## Case presentation

**Fig. 1:**
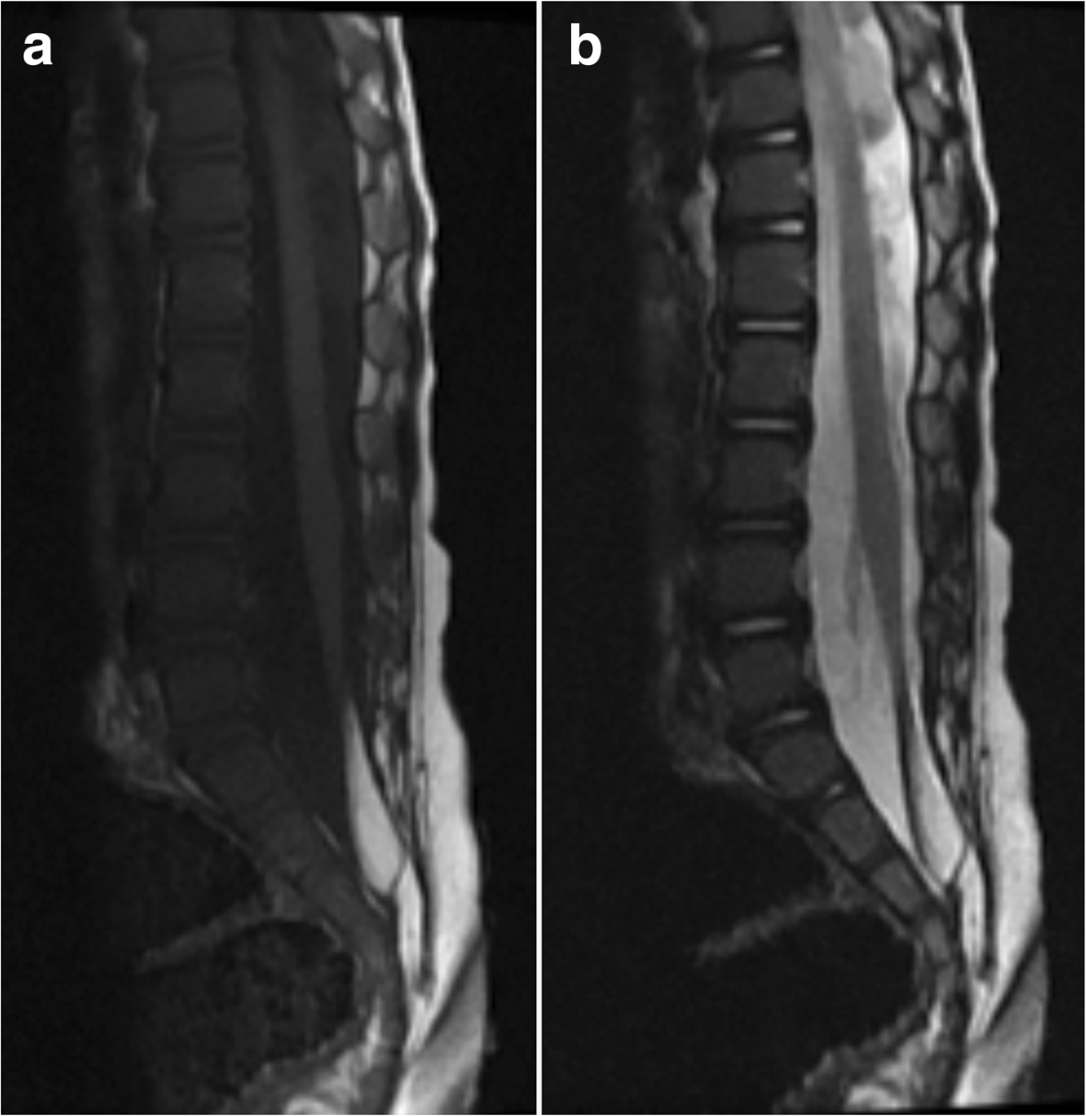
**a**. T1 weighted MR images demonstrating low conus, fatty filum, and sacral intradural lipoma **b**. T2 weighted MR images demonstrating low conus, fatty filum, and sacral intradural lipoma

A 2-year-old female was diagnosed with Sotos syndrome after genetic analysis demonstrating NSD1 mutation and 5Q35 microdeletion and she was under follow-up since birth. Besides the features of the syndrome, her main problem was relapsing urinary tract infections which were related to urinary retention due to neurogenic bladder. She was not able to urinate by herself and intermittent urinary catheterization was utilized by her mother as suggested. A urologist evaluating the child suspected from a spinal pathology and ordered a magnetic resonance imaging which showed a fatty filum terminale and a distal lipoma tethering the spinal cord between. The patient’s conus medullaris was at the level of the fifth vertebra and sacral space was filled with the lipoma (Fig. 1). At the admission, physical and neurological examination revealed spastic paraparesis in her lower extremities (Ashworth grade 2). Her head circumference was over 90 percentile and she had other features of the Sotos syndrome such as growth retardation, frontal bossing and hypertelorism. Although her problems may easily be related to the Sotos syndrome, the presence of a clear spinal cord tethering demonstrated in the MRI led us to the decision of a spinal exploration and untethering of the cord.

**Fig. 2:**
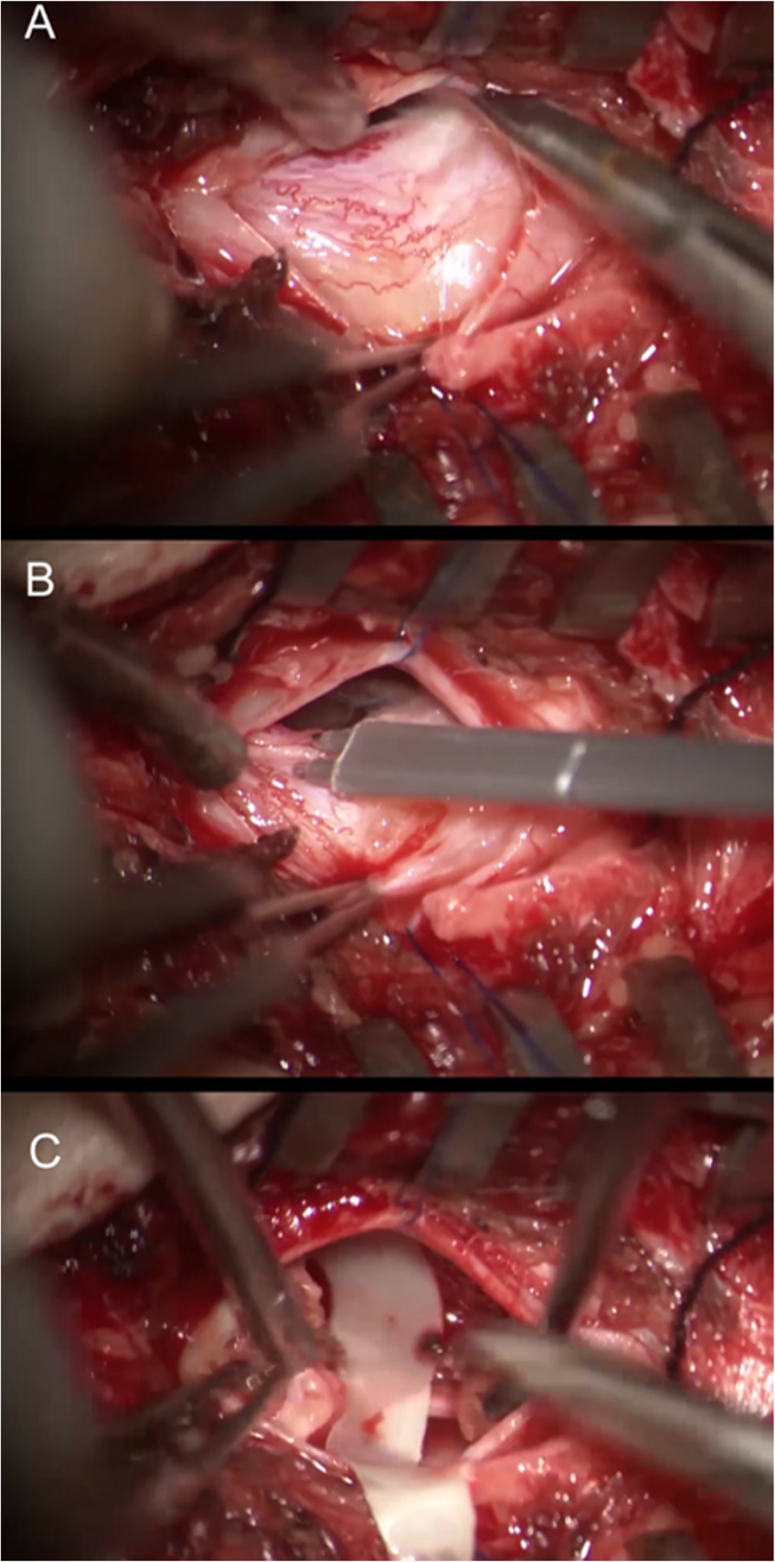
Intraoperative images taken through the surgical microscope. **a** Fatty and thickened filum terminale after dural opening **b.** Application of intraoperative neuro monitorization to spare adjacent rootlets. **c**. Sectioned filum terminale

A standard approach for tethered cord syndrome was utilized in this patient to untether the spinal cord. The operation was performed under intraoperative neuro monitorization and the thickened, lipomatous filum terminale was sectioned successfully (Fig. 2). The patient was discharged 3 days after without any additional neurodeficits and she is still under follow up for her spasticity and the status of urinary retention.

## Discussion and conclusion

Sotos syndrome is reported to occur in approximately 1/10,000–15,000 of births [5]. It is characterized by certain facial features. Early teething, high arching palate, pointed chin, prominent jaw, plethoric face, frontoparietal balding and macrocephaly are among those features. Additionally, kyphosis or scoliosis can also be seen in those patients however, there is no report in the previous literature associating Sotos syndrome with an intraspinal lipoma and tethered spinal cord.

Kurotaki et al. reported the underlying genetic abnormality of the Sotos Syndrome as mutations of the NSD1 gene which acts as a co-repressor or a co-activator [6]. NSD1 includes two nuclear receptor-interacting domains (NIDs). These ligand-binding area perform on part of nuclear receptors which characteristics of both co-activators and co-repressors. The chromosome 5q35.2 gene on the NSD1 gene likewise plays a role in these two tasks. The NSD1 gene makes directives for making a protein that functions as a histone methyltransferase. Histones are structural proteins that bind to DNA (deoxyribonucleic acid) and Histone methyltransferases enzymes are modified. Members of the NSD family have a key role in controlling cell growth and differentiation for this subgroup of SET domain proteins which called nuclear receptor-binding proteins. The NSD1 enzyme controls the activity of normal growth and development of these genes. Abnormality in NSD1 protein leads to uncontrolled overgrowth [6, 7].NSD1 microdeletion deletes genetic material from the part of chromosome 5. So NSD1 gene mutations cause an abnormally small, nonfunctional enzyme. And then NSD1 enzyme disrupts the normal activity of genes involved in growth and development because NSD1 gene plays a role in the development of important hormones such as steroids and thyroids required for growth. In the light of current studies, the relationship of these genes specific to phenotype has not been explained yet. However, disruption of proper growth as a result of deletion defect in these genes may cause this dysmorphic phenotype in Sotos syndrome. At the same time, uncontrolled growth may cause phenotype properties as well as results such as spinal lipoma.

However, it is not known exactly how a shortage of this enzyme during development leads to overgrowth, mental retardation and the other signs and symptoms of Sotos syndrome. We thought that this uncontrolled growth may have triggered the spina bifida and the formation of intradural lipoma tethering the cord.

Tethered cord syndrome and fatty filum terminale are rare neurological conditions. Fatty filum terminale or benign lipoma continuous to the spinal cord like a lipomyelomeningocele, in which a lipoma extrudes from the spinal canal underneath the lining of the spinal cord that covered by normal skin. In some cases, it may be the result of improper growth of the neural tube during fetal development, which is closely linked to spina bifida. Although tethered cord syndrome is associated with other syndromes such as sacral agenesis, VACTAREL syndrome, multiple pterygium syndrome (MPS) and Escobar syndrome [8], there is no previous report on the association of tethered cord syndrome with the Sotos syndrome.

We found only one study about spinal malformations in the Sotos syndrome. Lim et al. reported 8 patients with Sotos syndrome who had spina bifida and other spinal anomalies [9]. In their series, they report that all 8 patients had features of spina bifida. Although they do not clearly describe details of the spina bifida in their article we assumed that the described spina bifida was in a mild form of arcus fusion defects since they highlight that in none of their cases there were no thickened filum, tethered cord syndrome or dermal sinus tract requiring surgical intervention. So, we believe our case is the first one described in the literature that presents with Sotos syndrome and an additional tethered cord.

Sotos syndrome is a rare and complicated syndrome. Patients with this syndrome should be thoroughly evaluated for additional – surgery requiring – spinal pathologies.

## Data Availability

We obtained permission from the patient’s family to use all the materials for this case report and all materials used belong to the archive of our own clinic in this case report.

## Abbreviations

NSD 1: Nuclear receptor SET domain-containing protein 1
DNA: Deoxyribonucleic acid
VACTERL: Stands for vertebral defects, anal atresia, cardiac defects, tracheoesophageal fistula, renal anomalies, and limb abnormalities.
MPS: Multiple pterygium syndrome
